# Accelerated rifting in response to regional climate change in the East African Rift System

**DOI:** 10.1038/s41598-025-23264-9

**Published:** 2025-11-10

**Authors:** James D. Muirhead, Liang Xue, Robert Moucha, M. Keith Paciga, Emily J. Judd, Christopher A. Scholz

**Affiliations:** 1 School of Environment, University of Auckland, Auckland, New Zealand; 2 Department of Earth & Environmental Sciences, Syracuse University, Syracuse, USA; 3 Earth Sciences New Zealand, Auckland, New Zealand

**Keywords:** Climate sciences, Solid Earth sciences

## Abstract

Continental rifting is influenced by interactions between tectonic, magmatic, and surface processes, with the latter strongly dependent on regional climate. We test the role of regional climate variability on rift system behavior, by investigating fault slip rate changes in the South Turkana Basin (Lake Turkana Rift, northern Kenya) at the end of the African Humid Period. Throw rates on 27 faults examined during the African Humid Period (9,631–5,333 yr BP) and post-African Humid Period (5,333 yr BP–present) exhibit a mean 0.17 ± 0.08 mm/yr increase during the drier, post-African Humid Period. Numerical simulations reveal Coulomb stress changes from two loading sources that may explain these changes: (1) reduced vertical loading from a 100–150 m lake level drop, and (2) increased magmatic loading from enhanced mantle melt production due to reduced lake loading. An increase in magma flux of > 0.1 km^3^/kyr below the South Turkana Basin results in Coulomb stress changes exceeding those expected from a 100–150 m lake level drop. We provide the first empirical evidence of increased fault activity in response to climate-induced lake level changes in the East African Rift System over time scales of 10^3^–10^4^ years, and reveal that climate-tectonic interactions are enhanced in magmatically active rift systems.

**Supplementary Information:**

The online version contains supplementary material available at 10.1038/s41598-025-23264-9.

## Introduction

Interactions between magmatism, faulting and climate-induced surface processes play a critical role in controlling rift dynamics at divergent plate boundaries. For example, increased erosion rates driven by climate-induced changes in precipitation on rift flanks, and related sediment loading in the subsiding rift basin, increases maximum possible fault heaves and the overall lifespans of individual faults^1–3^. Similarly, glacial unloading in warming climates is thought to increase mantle melt production in Iceland and Yellowstone (USA)^4–7^, and alter stress fields around magma chambers^8,9^, which in turn can influence the mode of extensional strain accommodation at extensional settings (e.g., diking vs. tectonic faulting^10,11–13^). Dike-driven rifting can even be initiated due to pore-pressure changes associated with anomalously high rainfall^14^. Spectral analyses of sea-floor bathymetry also show systematic variations in fault-controlled topography that can be matched to orbital cycles and their effects on global sea-level^15^, although similar patterns can emerge without ocean water loading changes^16^.

It follows that climate-driven fluctuations in water levels in rift lakes, and hence changes in lake-loading, should have a profound impact on continental rift processes; however, the interactions between rift faults and varying lake levels are rarely examined or tested with empirically-derived data^17–20^. The East African Rift System represents an ideal locality for quantitatively examining changes in fault slip rates resulting from transitions to different climatic states. This continental rift system contains numerous large, deep lakes undergoing extensional processes and involving varying amounts magmatism^21–23^. The rift valley, filled in places by deep lakes, has experienced dramatic Plio-Pleistocene and Holocene hydroclimate fluctuations, resulting in lake-level changes on the order of hundreds of meters (e.g., Lakes Malawi, Tanganyika, and Turkana^24–27^). Indeed, cycles of lake-reservoir filling and emptying are shown to modulate crustal strength and associated seismogenesis^19,28^. Similarly, modelled Coulomb stress changes associated with climate-induced variations in lake loading in the Lake Malawi Rift support varying fault system behavior between different climate states^19^; however, no empirically-derived dataset has been presented to date that quantitatively demonstrates these predicted changes in fault slip. Here, we present the first quantitative evidence linking time-averaged fault slip rates to climate-driven lake level changes in the East African Rift System, by quantifying changes in fault slip rates as Lake Turkana in northern Kenya transitioned from high- to low-stand conditions at the end of the African Humid Period. These data highlight critical interactions between climate, surface processes, and magmatism that impact the evolution and architecture of a continental rift system.

## Results

**Fig. 1:**
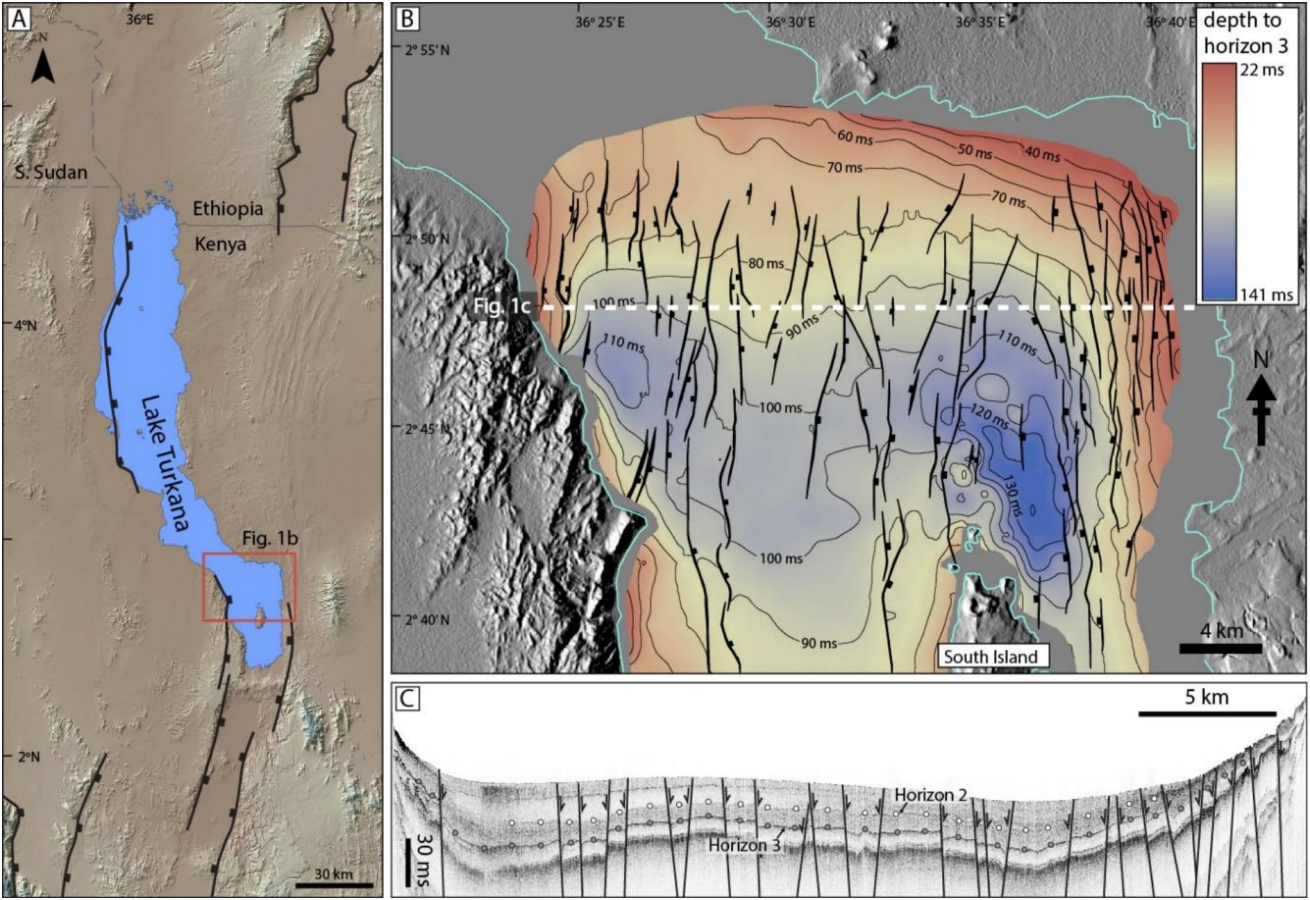
Structural setting of the study site. (**a**) Annotated SRTM DEM showing the Lake Turkana Rift of northern Kenya. The fault structure is simplified and used only to show the general rift pattern and position of modern border faults. (**b**) Structure contour map of the South Turkana Basin study site from Ref^36^. (**c**) Seismic profile Turk10-70 showing the fault structure and seismic horizons 2 (white-filled circles) and 3 (grey-filled circles) interpreted and mapped from Ref^36^ and used in this study.

The Lake Turkana Rift is situated along the relatively magma-rich Eastern branch of the East African Rift System, associated with the broader ~ 40 Ma Turkana Depression of the Kenya Rift^29–33^ (Fig. 1). The modern rift lake (Lake Turkana) is ~ 250 km-long and ~ 30 km-wide, with mean and maximum water depths of ~ 30 m and 120 m, respectively. The lake is situated within a region of active rifting and is bounded by a series of N-S-striking border faults, with rift extension and basin subsidence driven by displacements along these structures, and intra-rift faults below the lake^32,34–36^. Examination of GPS^37^ and subsurface fault system data^36^ reveal that Holocene extension in southern regions of the Turkana Depression is focused in a ~ 30 km-wide zone of axial faulting and magmatism in the South Turkana Basin (Fig. 1). Ref^36^ mapped the normal fault system in the South Turkana Basin north of South Island volcano (Fig. 1), utilizing over 1,100 km of high-resolution Compressed High Intensity Radiating Pulse (CHIRP) 2D seismic reflection data (subbottom penetration of ~ 35 m). Rates of faulting and rift extension were constrained from faulted offsets on dated seismic horizons, revealing that this region accounts of 3.5–5.8 mm/yr of rift extension, with extensional strain focused around the South Island axial volcano^36^.

Utilizing this same dataset^36^, we examined fault throw rates during two specific time periods: 9631–5333 yr BP, herein referred to as the late-African Humid Period, and 5333 yr BP–present, referred to herein as the post-African Humid Period. These chosen time intervals are based primarily on the mean ages of the strongest and most laterally continuous seismic reflections below the lakebed (Horizons 2 and 3; Fig. 1), rather than the exact timing of the end of the African Humid Period, which was a protracted event across Africa, with the end of the African Humid Period typically reported between 4000 and 6000 yr BP^25,38^. These time intervals (9631–5333 yr BP and 5333 yr BP–present) are, however, particularly relevant for Lake Turkana, as they occur either side of a distinct lake-level fall in Lake Turkana of 100–150 m occurring between 6000 and 4000 yr BP^25^, with the lake lowstand continuing to the present day.

**Fig. 2:**
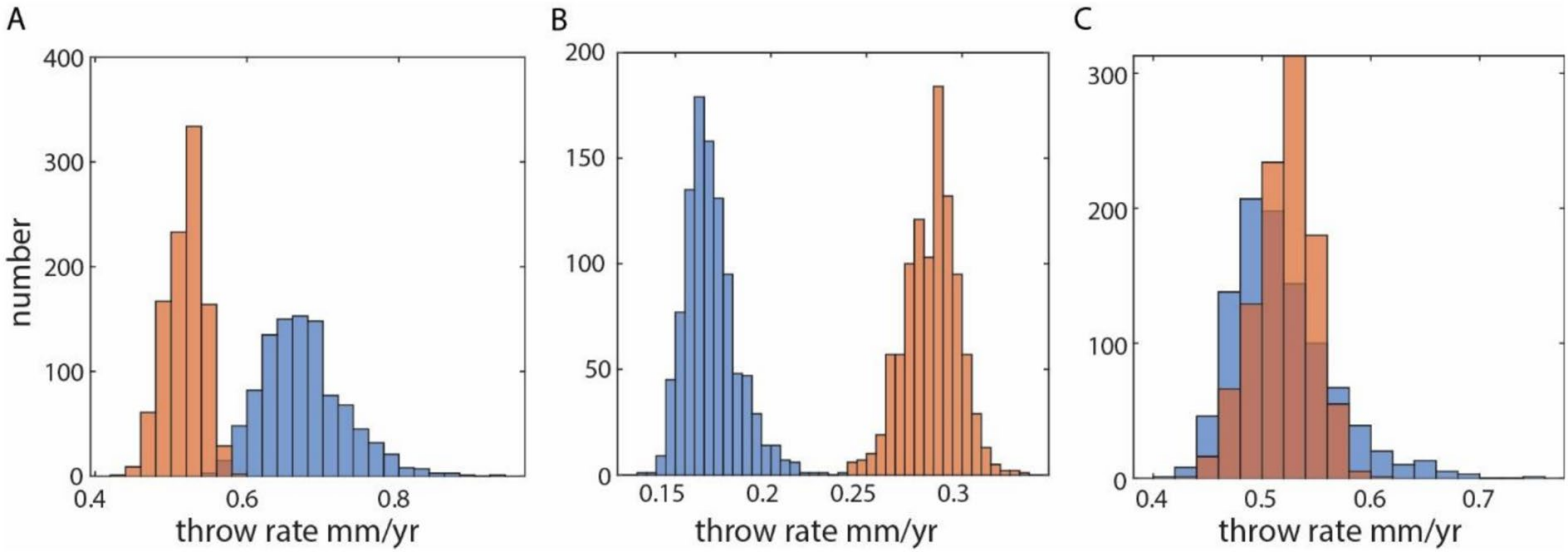
Examples of data extracted for three faults in this study. The range of possible throw rates during the African Humid Period (blue) and post-African Humid Period (orange) were acquired from 1000 Monte Carlo simulations. Results provide examples of faults showing a (**a**) reduction, (**b**) increase, and (**c**) no change in throw rate during the post-African Humid Period. The locations of all analysed faults and results of all simulations for 27 faults are shown in Supplementary Figs. S1–S5, and Supplementary Tables S1–S3.

We applied a Monte Carlo method that examines the range of possible fault throw histories in the South Turkana Basin based on the age and throw data for seismic horizons cut by 27 of the largest faults in the study region (Fig. 2, Fig. S1–S5) (see also Methods). Our analysis of the two most prominent seismic horizons reveals secular variations in throw rates in Lake Turkana during the Holocene. Changes in throw rates from the late- to post-African Humid Period range from − 0.15 to 1.16 mm/yr, with a mean change for all 27 faults of 0.17 ± 0.08 mm/yr (90% confidence). Of the 27 analyzed faults, 74% show an increase (to 90% confidence) in time-averaged throw rate in the post-African Humid Period (5,333 yr BP–present) (Table 1). By contrast, only 11% of the analyzed faults exhibit a decrease (to 90% confidence) in time-averaged fault throw rate during the post-African Humid Period (Table 1). Two distinct regions of the South Turkana Basin exhibit a pronounced increase in fault throw rate: (1) the western border fault region, and (2) the magmatic rift axis, where faults are aligned with South Island volcano (Fig. S6). The observed overall increase in time-averaged fault throw rate across the fault population, presented in Fig. 2 and Table 1, coincides not only with the end of the African Humid Period, but also major changes in lake level in Lake Turkana, where the lake level gradually reduced by ~ 100–150 m after the African Humid Period^25^.

**Table 1:**
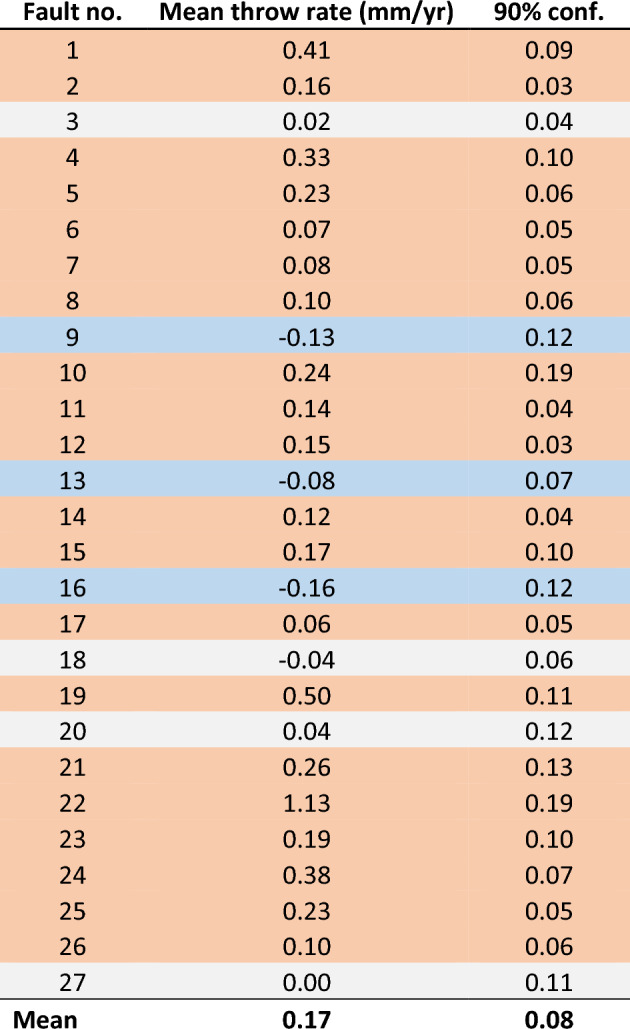
Changes in throw rate for all 27 faults.

Positive throw rates (orange) represent an increase in throw during the post-African Humid Period (to 95% confidence), whereas negative throw rates (blue) represent a decrease in throw during the post-African Humid Period (to 90% confidence). Grey faults show no discernible change

**Fig. 3:**
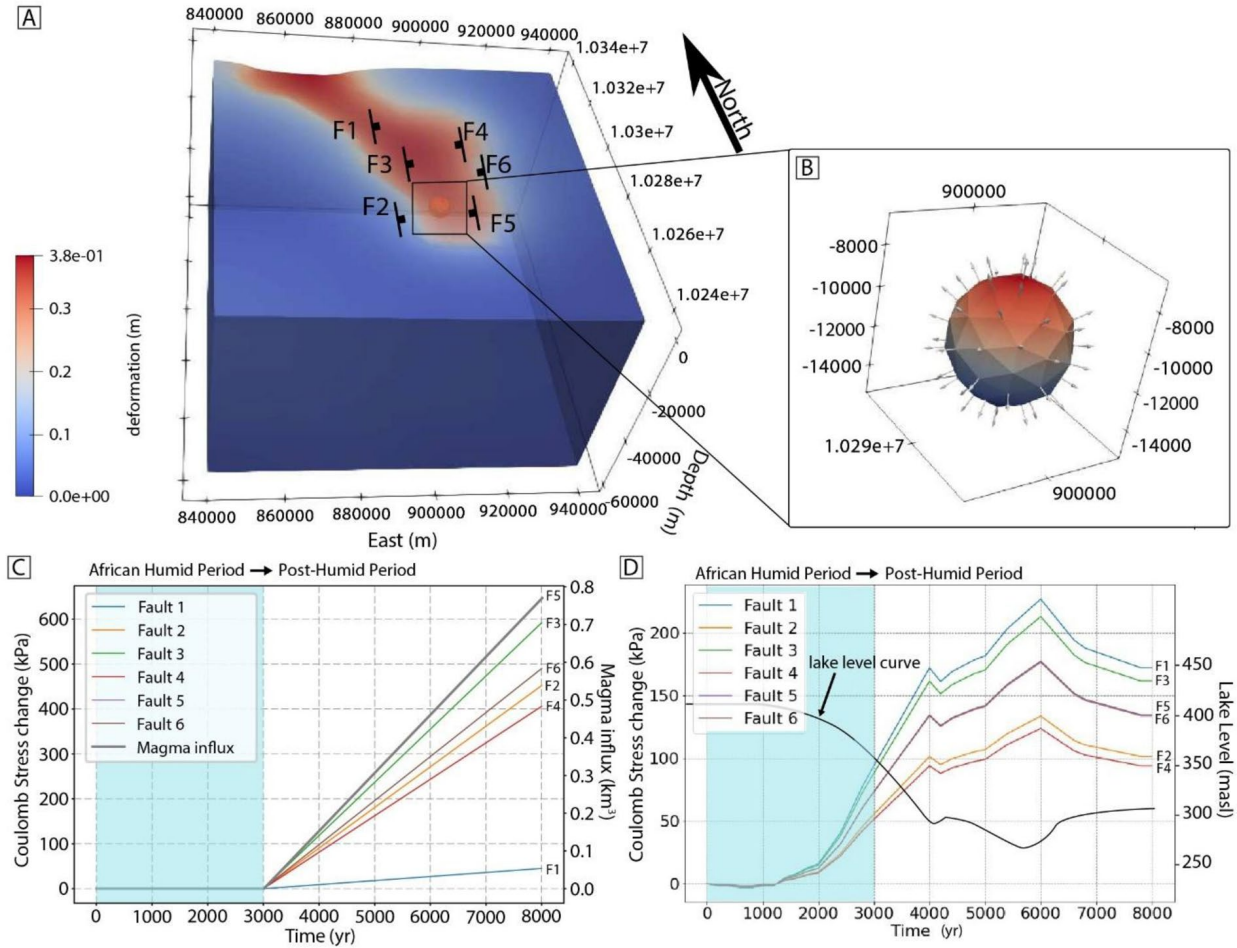
Pylith simulations testing the range of expected Coulomb stress changes associated with varying magmatic and lake loads. (**a**) Finite element model domain showing the elastic response to a lake water load added to the domain surface, and black lines showing the positions of 6 hypothetical rift faults. (**b**) 3 km radius magma chamber, centered at 10 km depth, included for model runs shown in C. (**c**) Coulomb stress changes on faults shown in A, associated with the inflation of the magma chamber in B at a rate of 0.154 km^3^/kyr for 5,000 years (i.e., approximate current timeframe of the post-African Humid Period). (**d**) Coulomb stress changes on faults shown in A, associated with lake level variations (from Ref^25^) estimated for the Post-African Humid Period. Note that C and D are forward models, and that the Post-African Humid Period initiates at 3 kyr and is modelled for a total of 5 kyr.

To further explore the physical processes driving this increase in fault throw rate, the feedbacks between climate-driven lake level changes and mechanical rift processes were investigated using a series of simulations in PyLith (see Methods). Coulomb stress changes at various fault localities across the South Turkana Basin respond to two loading mechanisms: (1) lake level fluctuations and (2) magma chamber inflation beneath South Island volcano (Fig. 3). Increased pressure in the magma chamber below South Island volcano is expected as a result of enhanced mantle melt production driven by decompression melting in response to lake unloading during low-stand lake phases. For example, recent numerical studies of mantle melting in response to reduced lake loads predict enhanced mantle melt volumes on the order of 0.07–0.34 km^3^ per thousand years for a 50 km-long rift basin during periods of reduced lake loading^39^. Additional magma volumes of, for example, 0.154 km^3^/kyr fed into the underlying South Island magma system during the lake lowstand period (i.e., last 5000 years) should produce excess magma pressures that are estimated to affect the local stress state surrounding the South Turkana Basin fault system. Indeed, Coulomb stress changes as high as 650 kPa are expected on the South Turkana Basin faults during the last 5000 years for a 3 km radius chamber, centered at 10 km depth^40^, with an excess magma flux of 0.154 km^3^/kyr (Fig. 3). By contrast, the elastic response of South Turkana Basin to a reduction in 100–150 m of lake loading would result in Coulomb stress changes of 95–230 kPa for South Turkana Basin faults (Fig. 3). Although both modeled loading sources are shown to drive Coulomb stress changes that promote normal faulting during lake lowstand periods in the South Turkana Basin, these results suggest the dominant source of loading is more likely from the underlying magma chamber (Fig. 3).

## Discussion

Our results provide robust empirical support for changing fault behavior in Lake Turkana at ~ 5.3 kyr BP, coinciding with changing regional climate across East Africa. Importantly, the African Humid Period impacted both terrestrial and aquatic environments across large swaths of East Africa in the early- to mid-Holocene^41–44^. Many of these lake systems are contained within subsiding rift basins of the East African Rift System, such as the Lake Turkana Rift, which also exhibits Holocene lake level changes associated with this regional climate event^25^.

Surface loads associated with these climatic changes are primarily provided by both sediment influx and lake water volumes, with the loading forces from both sources more likely to reduce overall in response to a shift to a drier climate, although sediment loads may become more concentrated in basin depocenters during lake lowstands^45,45^. Dated sediment cores across Lake Turkana show Holocene sedimentation rates ranging 0.6–2.4 mm/yr^25^, with a ~ 60% reduction in the rate of sedimentation during the post-African Humid Period observed in cores that cover the entire Holocene (i.e., core 46P of Ref^25^). Assuming a conservative mean sedimentation rate reduction of 1.5 mm/yr across Lake Turkana between the African Humid Period and post-Africa Humid Period, we estimate a total sediment thickness “deficit” of 7.9 m since 5,333 yr BP, which is a small value compared to the recorded ~ 100 to 150 m reduction in lake level^25^. Given this order of magnitude contrast it is likely that variations in lake loading will have a greater effect on rift processes on 10^2^–10^3^ year timescales, as the reduction in water loading exceeded sediment loading by an order of magnitude during the post-African Humid Period; therefore, we only model lake loading-related stress changes (rather than sediment loading) during the post-African Humid Period analyzed in this study.

Predicted Coulomb stress changes associated with lake unloading are shown in Fig. 3. These analyses reveal that the climate-induced lake level reduction in Lake Turkana likely would have produced favorable conditions for enhanced normal faulting, with predicted Coulomb stress changes ranging 50–650 kPa. Similar observations and modeling results showing increased fault activity in response to surface mass unloading have been documented for other continental extensional systems globally^18,46,47^, as well as in convergent tectonic settings^48^ and even passive margins^49^. Within the Basin and Range Province, flexural unloading from climate-induced deglaciation events is demonstrated to reduce horizontal stresses and increase rates of faulting, which, depending on the viscosity of the lithospheric mantle and asthenosphere, should exhibit lag times of hundreds to thousands of years^46,47^ and similar to the observational window of our dataset. In eastern Canada, increased faulting is predicted during post-glacial unloading periods^49^ and, similarly, the removal of sedimentary loads in the eastern Andean Plateau^50^ and Taiwan^48^ has been shown to enhance the likelihood of thrust faulting.

Although Coulomb stress changes from lake unloading are expected to promote normal faulting in the South Turkana Basin, magma-driven processes, including the indirect effects of lake-unloading on excess mantle melt production and elevated crustal magma pressures, are expected to further enhance the likelihood of normal faulting. In fact, excess mantle melt production may even be the primary driver of enhanced fault activity during drier climate states. Phase equilibria studies of fluid inclusions from Quaternary volcanic deposits at South Island volcano support the presence of a mid-crustal magma reservoir in the South Turkana Basin (~ 12 km depth^40^). The size of the magma chamber, combined with additional melt flux during lake lowstands, could generate magma pressures causing Coulomb stress changes up to 650 kPa since the onset of the post-African Humid Period (Fig. 3). Additionally, the region exhibiting the greatest increase in fault throw rate occurs along the magmatic rift axis within the zone of faults aligned with South Island volcano (Fig. S6), which is inferred to have experienced Holocene volcanic activity and played a key role in driving the current phase of magmatic rifting in the South Turkana Basin^36,40,51^. In all, these results underscore the role of active magmatism in enhancing climate-tectonic interactions in continental rift systems.

Failure of the magma chamber in response to excess magma pressure will also result in dike-rifting events like those recorded in other magmatic rift zones such as Afar and Iceland, which are shown to initiate normal faulting at shallow crustal depths (upper few km)^13,52^. Indeed, dike-driven rifting in magmatic rift zones in Hawaii can be triggered by comparatively small reductions in stress compared to those predicted for Lake Turkana, such as hydrostatic pressure changes related to increased rainfall^14^. Thus, increased melt production and magma supply into the rift axis is expected to both (1) induce magma chamber pressures that favorably load faults, and (2) increase the frequency of dike intrusion events and further amplify associated normal faulting within this region of magmatic rifting. Although the latter of these processes could not be modelled in this study, it is likely important for enhancing shallow fault activity during lake lowstands.

**Fig. 4:**
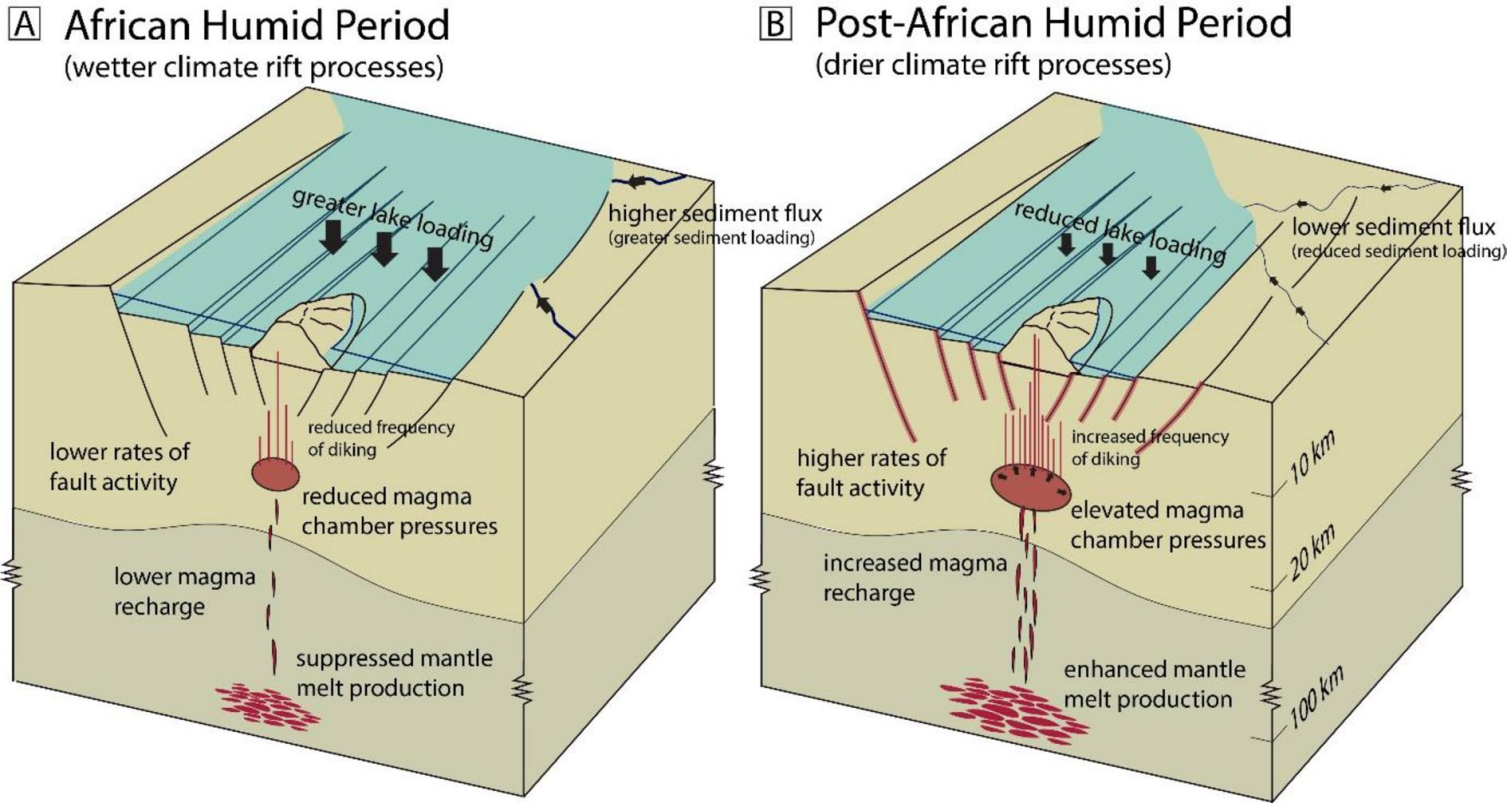
Conceptual illustration of the range of proposed processes driving increased fault activity in the Lake Turkana Rift in response to changing climatic conditions associated with the transition from the (**a**) African Humid Period to (**b**) the post-African Humid Period.

Figure 4 provides a conceptual illustration of the various processes that drive enhanced rates of normal faulting during drier climate episodes in the Lake Turkana region, with implications for magma-rich rift systems subject to lake loading in the East African Rift System (e.g., Main Ethiopian Rift). Here, the transition to a more arid climate state reduces hydrological inputs into the rift lake system, resulting in significant lake level drops (100s of meters) occurring over 100s to 1,000s of years. These climate shifts also diminish overall sediment inputs and their associated loads. The stress state associated with relaxation of the crust from a reduction in lake loading creates favorable conditions for normal faulting^19^ (Fig. 4), while mantle (decompression) melting is also enhanced from the associated pressure reduction in the lithosphere below the rift^39^. We expect this enhanced melting to result in a higher flux of mantle melt into the axial magmatic segment below the South Turkana Basin^40^, where fault loading from an overpressured magma chamber and axial diking leads to increased normal faulting rates in magmatically active regions like the Lake Turkana Rift.

Our study provides the first empirical dataset illustrating changes in time-averaged fault slip rate associated with changing climate regimes in East Africa. The East African Rift System exhibits a number of rift basin lakes in both magmatic (e.g., Lake Magadi, Kenya rift) and magma-poor regions (e.g., Lake Malawi Rift), which were impacted at various times by an evolving Quaternary climate^24,53^. The Main Ethiopian Rift records both tectonic- and hydroclimate-driven variations in lake levels on the order of 100s of m (e.g., Lakes Abiyata, Ziway, and Shala)^54–56^. Here, climate-driven lake level changes have been correlated with changing magmatic carbon release within associated hydrothermal systems^57^; however, our findings suggest that further evaluation of the impact of climate-driven lake level change on both the magmatic and tectonic system is merited. Similarly, a 70 kyr-long depositional hiatus is observed in the latter Pleistocene over large areas of Lakes Tanganyika and Malawi, during which lake levels fell by ~ 500–600 m^24^. Our results suggest these lake level variations can affect crustal stress states that impact fault system behavior, as well as magmatic processes that further modulate rift faulting. Climate, therefore, may influence strain localization, while possibly affecting the style of rifting (e.g., magmatic vs purely tectonic strain accommodation) as continent rifts evolve towards sea-floor spreading. Finally, we propose that the impact of climate on fault system behavior is more pronounced in rift systems experiencing high rates of magmatism.

## Supplemental methods

### Estimating time-averaged fault throw rates

Lake Turkana is a 250 km-long by ~ 30 km-wide zone of active rifting, associated with the broader ~ 40 Ma Turkana Depression of the Kenya Rift^32,33^ (Fig. 1). To better understand fault activity within the southern region of this lake (Fig. 1), we utilized over 1,100 km of high-resolution Compressed High Intensity Radiating Pulse (CHIRP) 2D seismic reflection data (subbottom penetration of ~ 35 m), integrated with a suite of Kullenberg piston cores collected in 2009–2011^25^. Three of these cores were radiocarbon dated by Ref^25^ to generate a sediment age model (later adapted by Ref^36^). These data allow us to constrain a Holocene history of fault activity in the South Turkana basin.

Syn-depositional (growth) fault analyses were used to understand the history of fault slip^58,59^. Syn-depositional faults gradually accumulate throw (*T*) during deposition of syn-rift sediments, such that fault displacements at the surface equal zero and gradually increase with depth. The time-averaged fault throw rate (*T*_s_, fault throw rate average over multiple surface rupture events) between two different time intervals (*t*_x_ and *t*_y_), represented by dated seismic horizons, can be estimated using:

1 $${T}_{s}=\frac{{T}_{x}-{T}_{y}}{{t}_{x}-{t}_{y}}$$

To measure fault throws in the South Turkana basin, we measured vertical offsets between two dated seismic horizons that can be mapped confidently below the lake using the CHIRP seismic reflection dataset (Fig. 1). Our data include only faults where clearly observable and measurable offsets can be identified on both horizons across individual faults observed in the seismic profiles. We model (discussed below) only the maximum throws observed along any one fault. Adopting a minimum fault throw cut-off of 1 ms, our analyses allow us to resolve time-averaged throw rates at locations of maximum throw on 27 faults in the study region.

We examine fault throw rates during two specific time periods: 9631–5333 yr BP, referred to as the late-African Humid Period, and 5333 yr BP–present, referred to as the post-African Humid Period. These chosen time intervals are based primarily on the mean age of the strongest and most laterally continuous seismic reflections below the lake, rather than the exact timing of the end of the African Humid Period, which was a protracted event across Africa with the end of the African Humid Period typically reported between 4,000 and 6,000 yr BP^25,38^. These time intervals (9,631–5,333 yr BP, 5333 yr BP–present) are however particularly relevant for Lake Turkana. Specifically, they occur either side of a distinct change in lake-level in Lake Turkana of  > 100 m occurring over a ~ 2,000 year time period from 6,000 to 4,000 yr BP^25^, with the lake lowstand continuing to the present day. Radiocarbon ages assume a reference age of 1950 CE; therefore, given that the analysed seismic data were collected in 2010 CE, we adopt ages of 9,631 and 5,333 yr BP for our fault slip analyses, with the “present day” considered to be 2010 CE^36^.

Our analyses were designed to discern changes in time-averaged fault throw rates over millennial timescales. Given the inherent age and throw uncertainties, a range of throw-time (*T-t*) paths are possible for each fault. To address this issue, we applied a Monte Carlo method that examines the range of possible fault throw histories for the acquired *T*-*t* data on each fault. Consistent with the method of Ref^60^, we performed 1000 simulations of possible *T-t* paths on throw-time plots (Figs. S1–S5), which were defined by *T-t* envelopes constrained by previously modelled ages of the mapped seismic horizons, and their uncertainties, as well as a range of possible throw values, assuming seismic velocities ranging 1470–1510 ms for the shallow saturated sediments (< 35 m depth from the water bottom)^36^. For each of the 1000 simulations, we recorded the time-averaged throw rates for our two time-periods of interest (late-African Humid Period and post-African Humid Period) (Fig. 3, Figs. S1–S5; Table S1–S2). We also recorded the change in throw rate between each time-period for each simulation, by subtracting the estimated throw rate for the post-African Humid Period by the throw rate for the late-African Humid Period (Table S3). Changes in throw rate presented for each fault (Table 1) represent the mean value of all 1000 simulations, as well as the 2 sigma standard error. Positive changes in throw rate correspond to an increase in throw rate during the post-African Humid Period.

### Numerical modelling

To approximate the changes to the subsurface stress field in the Lake Turkana region resulting from the inflation of a magma chamber and climate-related lake loading, we ran a series of 3D finite element simulations. The computations were conducted using an open-source FEM quasi-static modeling code PyLith 2.2.2^61^ from Computational Infrastructure for Geodynamics (CIG). A 3D model was meshed with the software Coreform Cubit in tetrahedral elements. The model domain was 100 × 100 × 80 km, with an average cell size of approximately 2 km. The Earth’s structure was derived from a homogeneous, isotropic, linear elastic material of the preliminary reference Earth model (PREM, Dziewonski and Anderson^62^) (Table S4).

The purpose of our modelling approach was to isolate the possible impact of each process (magma chamber inflation and climate-related lake loading) on faulting, and their relative contributions. Consequently, our models do not couple the effects of lake unloading and magma chamber inflation (see Fig. S8 for an example of a coupled model). Similarly, as our focus was to isolate the additive effects of vertical lake loading and magma inflation processes over a relatively short time period, the model does not include extension as a boundary condition, and hence we assume our results are not sensitive to rift opening rates.

Our model includes surface loading from lake level fluctuations based on the lake level curve of Ref^25^ and loading from a magma chamber at 10 km depth with a radius of 3 km. The depth of the chamber is based on the melt-inclusion study of Ref^40^, which shows that magmas below South Island likely pond between ~ 9 and 12 km depth, though perhaps as deep as 15 km. Thus we conservatively adopt a 3 km radius for the magma chamber, which ultimately makes the modelled stress state less sensitive to volumetric changes in the magma chamber.

We prescribe lake-level variations as a time-dependent surface load applied uniformly over a fixed Lake Turkana planform (modern shoreline):

2 $$\Delta P(t) \, = \rho_{w} \cdot{\text{g}}\cdot \, \Delta h(t)$$

where *P* is hydrostatic pressure, ρ_*w*_ is density of water, g is gravitational acceleration, and ℎ is lake water depth. This first-order approach neglects lateral variation in bathymetry and shoreline migration during drawdown; therefore, the effects of the spatial variability of the unloading (e.g., stronger effects on rift-floor faults directly beneath the former deep basin) are not captured. However, because all faults analyzed in this study are currently situated below the lake, and the lake is currently in its lowstand phase, all faults have likely experienced the same 100–150 m change in lake loading during the post-African Humid Period^25^ and therefore their response should be similar^63^.

The lake water load on the top surface and chamber inflation is imposed as the Neumann boundary condition. The displacements perpendicular to the lateral and bottom model boundaries are fixed to zero and the displacements parallel to these boundaries are not constrained (details follow the methods of Ref^64^), and the magma chamber surface is an inner boundary with inflation traction as a Neumann boundary condition. Note that the Neumann boundary condition is time-dependent, where surface loading is from lake level variations and the inner boundary is varying based on magma influx. Our models are quasi-static and thus do not account for dynamic effects that may influence the final fault distribution. The magma inflation started at 3 kyr and the magnitude of inflation traction is dependent on the magma flux (Fig. 2b).

We assume that intra-rift faults around the South Turkana Basin are north–south striking, dip-slip normal faults, consistent with data from Ref^36^. To model the effects of loading on these faults, we simplify their geometry by representing each fault as a straight, planar surface. Loading derived stress tensors, from changes in lake levels and/or the magma chamber, are then projected onto these simplified fault planes (details follow the methods of Ref^64^). The projection is based on the fault’s location and its specific orientation parameters (strike, dip, and rake). Subsequently, Coulomb stress change (∆CF) on the fault planes is calculated using the equation

3 $$\Delta {\text{CF}} = \, \Delta \tau \, + \, \mu \Delta \sigma$$

where ∆τ and ∆σ represent changes in shear and normal stress, respectively, and µ is the frictional coefficient (here we use a µ value of 0.4^65^). The Coulomb stress change is calculated in our model at a depth of ~ 2.5 km for simplification. We also ran sensitivity analyses testing Coulomb stress changes for a range of magma fluxes, and for a coefficient of friction of 0.6^66,67^.

The governing equations for the quasi-static materials can be derived from the momentum balance conservation equations. Pylith solves the elasticity equation including the inertial terms:

4 $$\nabla \cdot {\sigma }_{ij}+{f}_{i}=\rho \frac{{\partial }^{2}{u}_{i}}{\partial {t}^{2}}i,j=x,y,z,$$

5 $${\sigma }_{ij}{n}_{j}={T}_{i}on{S}_{T},$$

6 $${u}_{i}={u}_{i}^{0}on{S}_{u},\text{ and}$$

7 $${R}_{ki}$$

where ${\sigma }_{ij}$ is the stress tensor (positive for tension), ${f}_{i}$ is the body force acting in the direction of gravity, $\rho$ is the bulk density, and t is the time. We used normal vector ${n}_{j}$, traction $T$ on surface ${S}_{T}$, displacement ${u}_{i}^{0}$ on surface ${S}_{u}$, and slip ${d}_{k}$ on fault surface ${S}_{f}$, where the tractions and fault slip are in global coordinates, and ${R}_{ki}$ is a rotation matrix that transforms the global coordinate system to the fault coordinate system.

## Supplementary Information

Supplementary Information 1.

Supplementary Information 2.

Supplementary Information 3.

Supplementary Information 4.

Supplementary Information 5.

## Data Availability

The fault data used in this study can found at Figshare (10.6084/m9.figshare.29577815) and the Matlab code used for analyzing these fault data at Zenodo (10.5281/zenodo.15953260). The finite element modeling is performed using PyLith^61^, available from CIG (https://geodynamics.org/resources/pylith). The PREM model is accessible from IRIS database (https://ds.iris.edu/spud/earthmodel/9991844). Results of the Mote Carlo simulations for 27 faults can be found in Supplementary Tables S1–S3.
