# Supplementary material for: Accelerated rifting in response to regional climate change in the East African Rift System
Source: Sci Rep. 2025 Nov 10;15:38833. doi: 10.1038/s41598-025-23264-9 (PMC12603047; doi:10.1038/s41598-025-23264-9)
Supplement: Supplementary file 1 — Supplementary Information 1. [file 41598_2025_23264_MOESM1_ESM.pdf]

SUPPLEMENTARY FIGURES

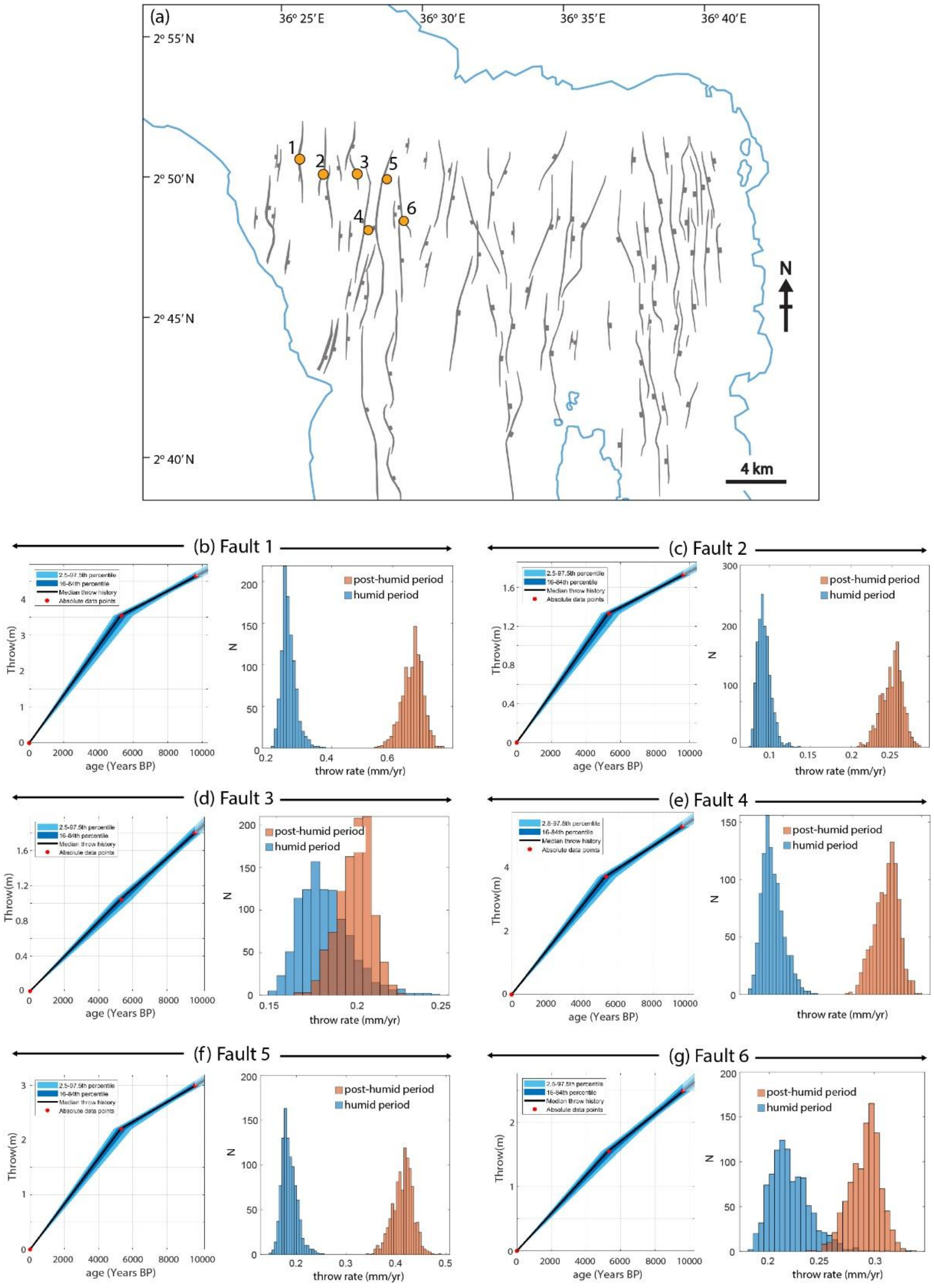

Supplementary Figure S1: Result from 1000 Montecarlo simulations for Faults 1 to 6 in the study region. Inset map showing the locations along each fault where throw measurements were collected for these analyses. For each fault (b-g), the plot on the left shows the fault throw history, whereas the plot on the right is a histogram showing the distribution of simulated throws rates during both the post-African Humid Period and African Humid Period.

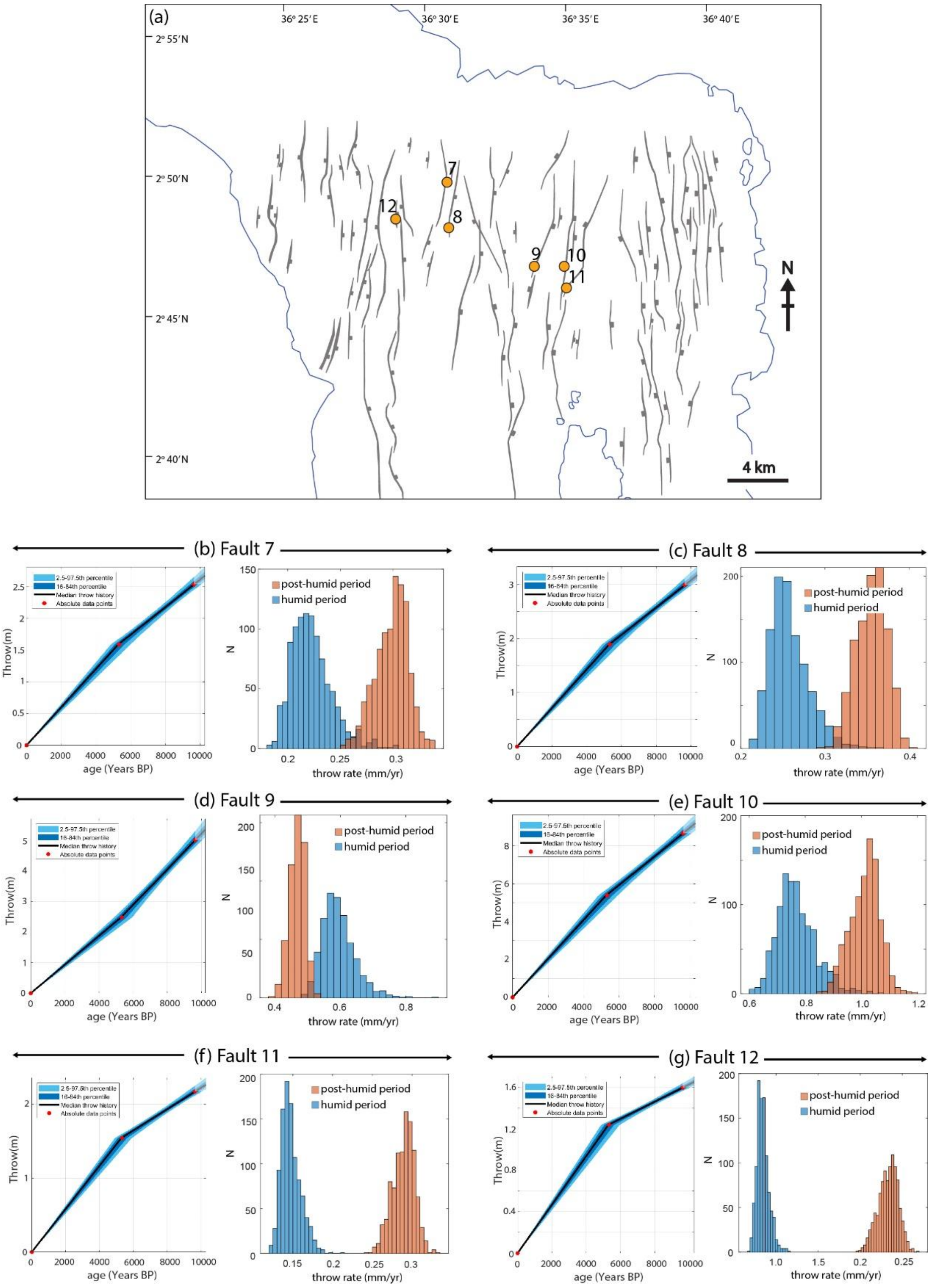

Supplementary Figure S2: Result from 1000 Montecarlo simulations for Faults 7 to 12 in the study region. Inset map showing the locations along each fault where throw measurements were collected for these analyses. For each fault (b-g), the plot on the left shows the fault throw history, whereas the plot on the right is a histogram showing the distribution of simulated throws rates during both the post-African Humid Period and African Humid Period.

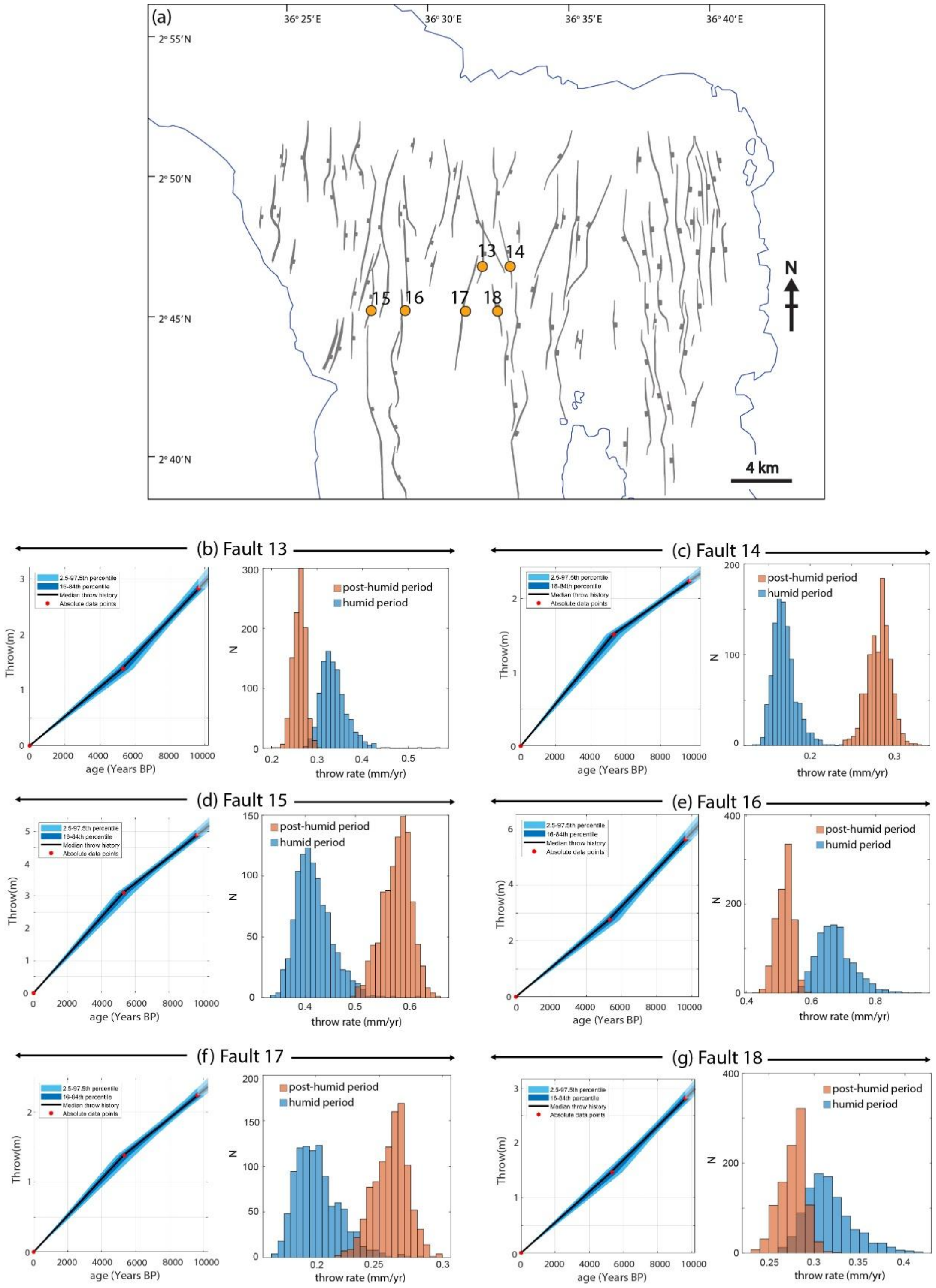

Supplementary Figure S3: Result from 1000 Montecarlo simulations for Faults 13 to 18 in the study region. Inset map showing the locations along each fault where throw measurements were collected for these analyses. For each fault (b-g), the plot on the left shows the fault throw history, whereas the plot on the right is a histogram showing the distribution of simulated throws rates during both the post-African Humid Period and African Humid Period.

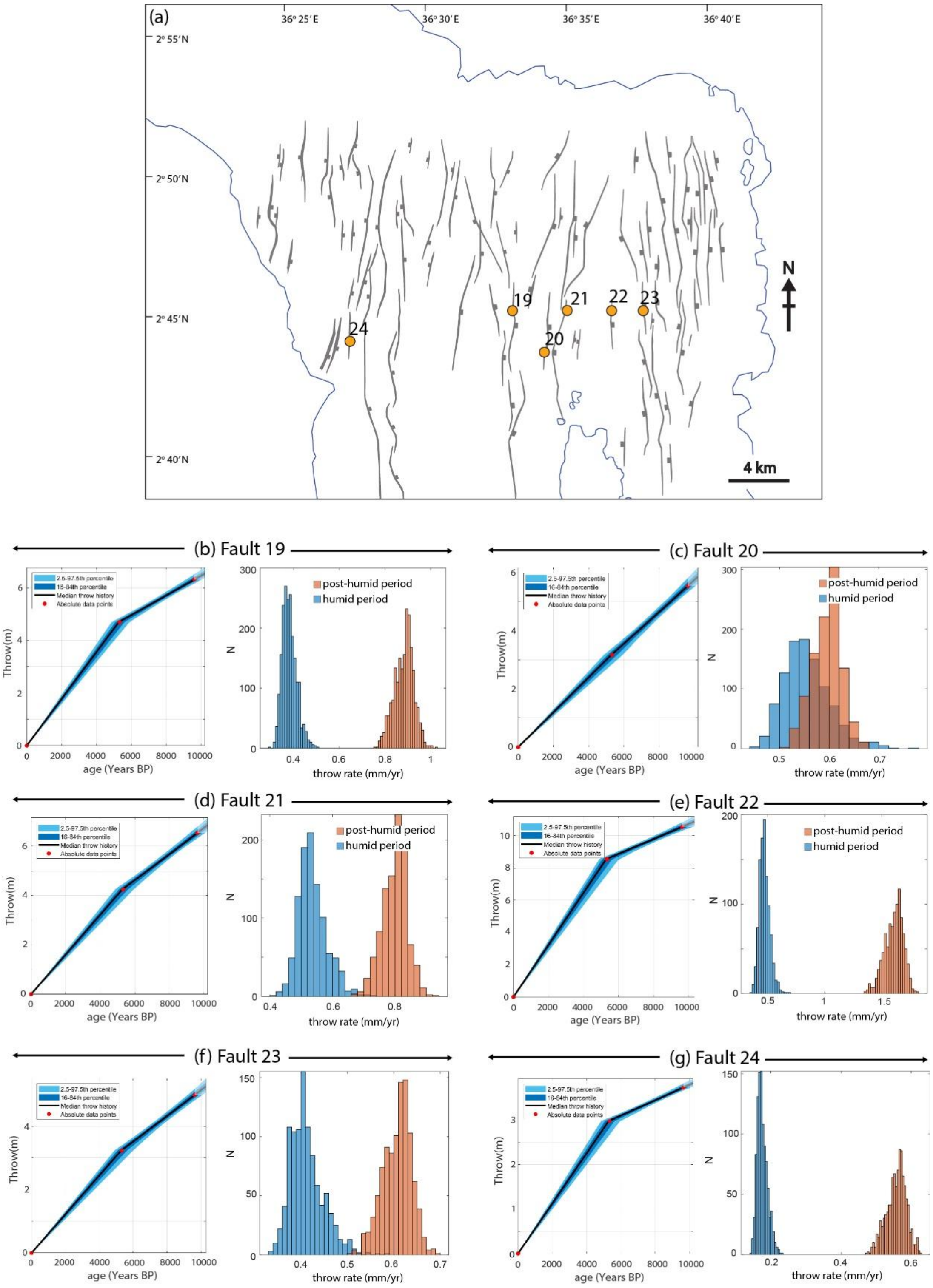

Supplementary Figure S4: Result from 1000 Montecarlo simulations for Faults 19 to 24 in the study region. Inset map showing the locations along each fault where throw measurements were collected for these analyses. For each fault (b-g), the plot on the left shows the fault throw history, whereas the plot on the right is a histogram showing the distribution of simulated throws rates during both the post-African Humid Period and African Humid Period.

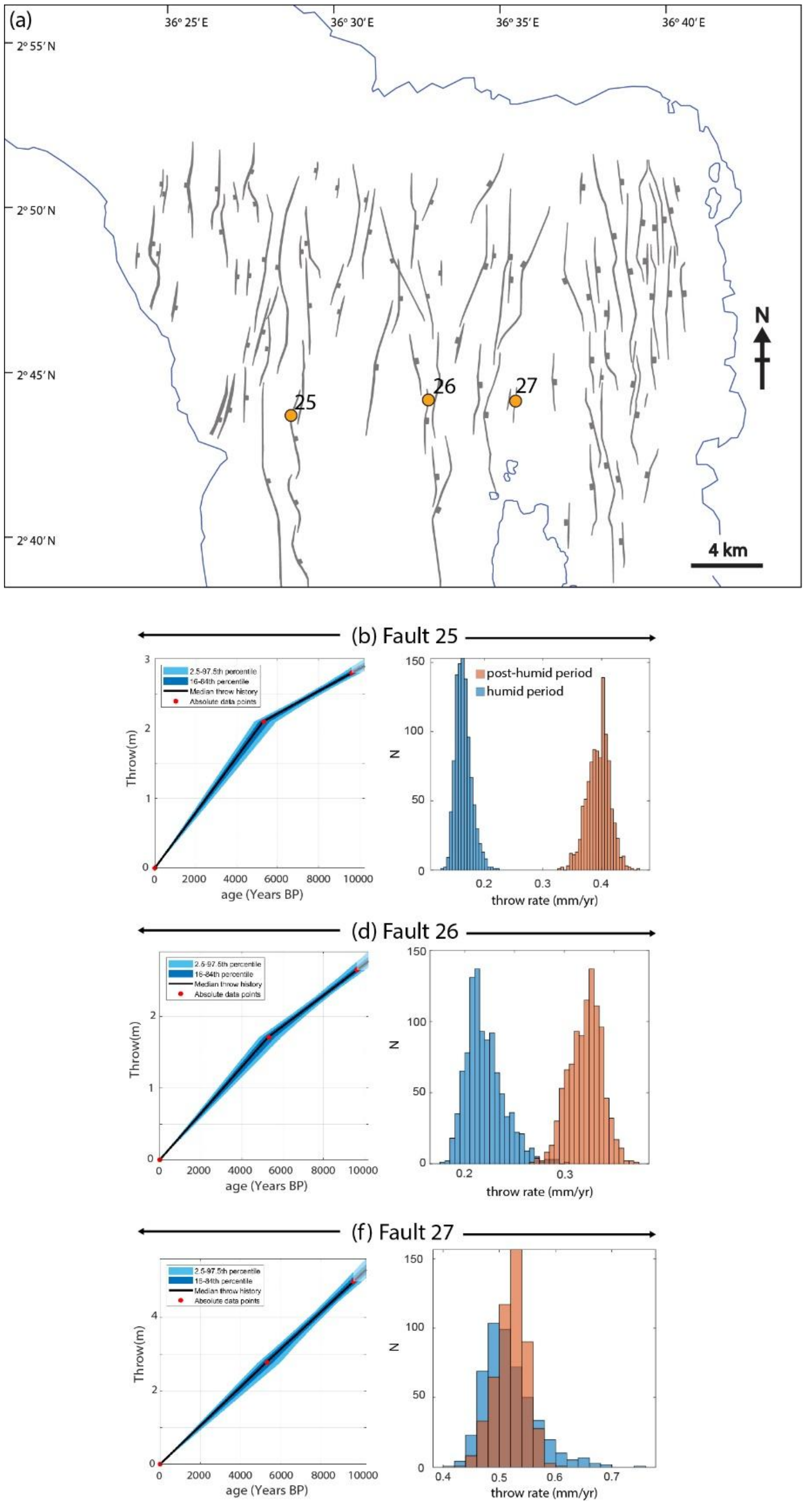

Supplementary Figure S5: Result from 1000 Montecarlo simulations for Faults 25 to 27 in the study region. Inset map showing the locations along each fault where throw measurements were collected for these analyses. For each fault (b-g), the plot on the left shows the fault throw history, whereas the plot on the right is a histogram showing the distribution of simulated throws rates during both the post-African Humid Period and African Humid Period.

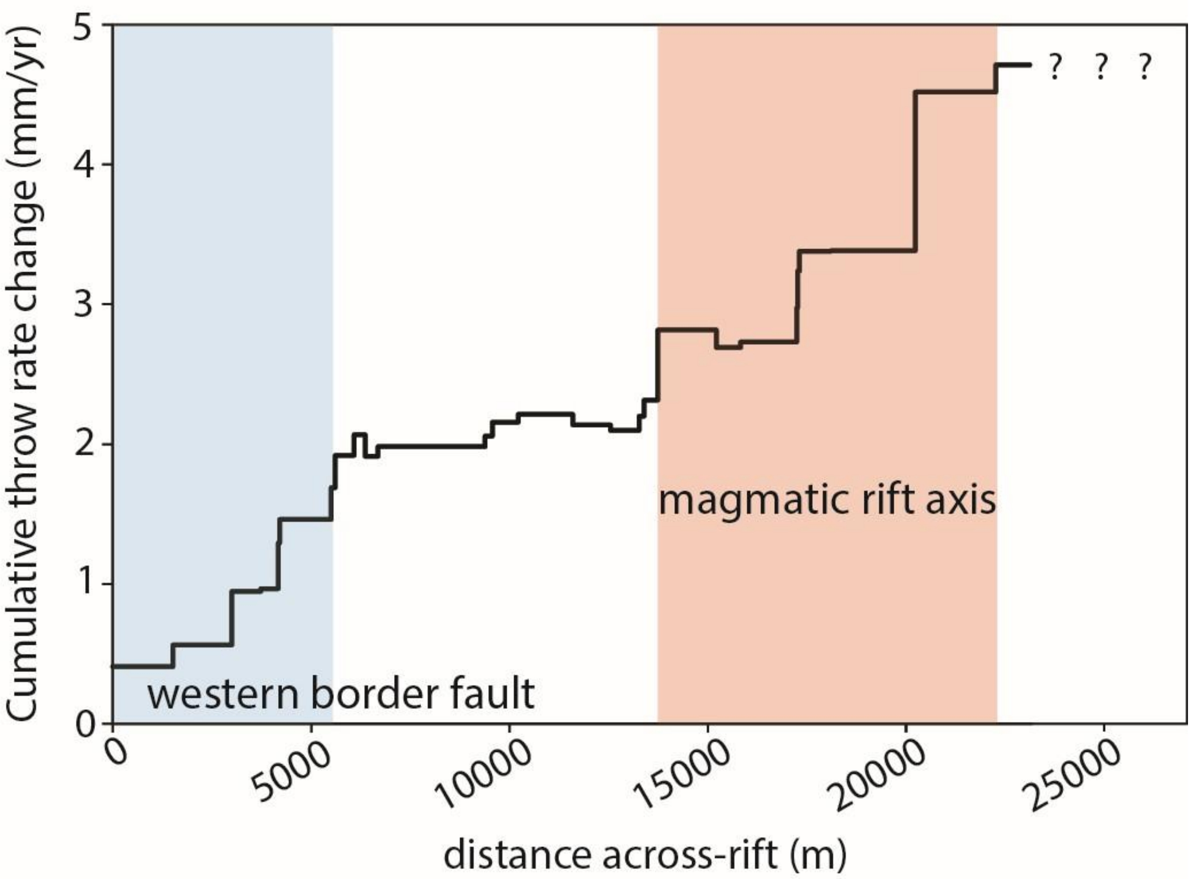

Supplementary Figure S6. Across-rift changes in throw rate change in the South Turkana Basin, based on fault analyses shown in Figures S1-S5. The cumulative throw rate is summed from west to east across the basin. The locations of each fault within the transect is shown in Figure S7. The “magmatic rift axis” refers to faults the are found with the zone of inferred magmatic rifting[37] aligned with South Island volcano.

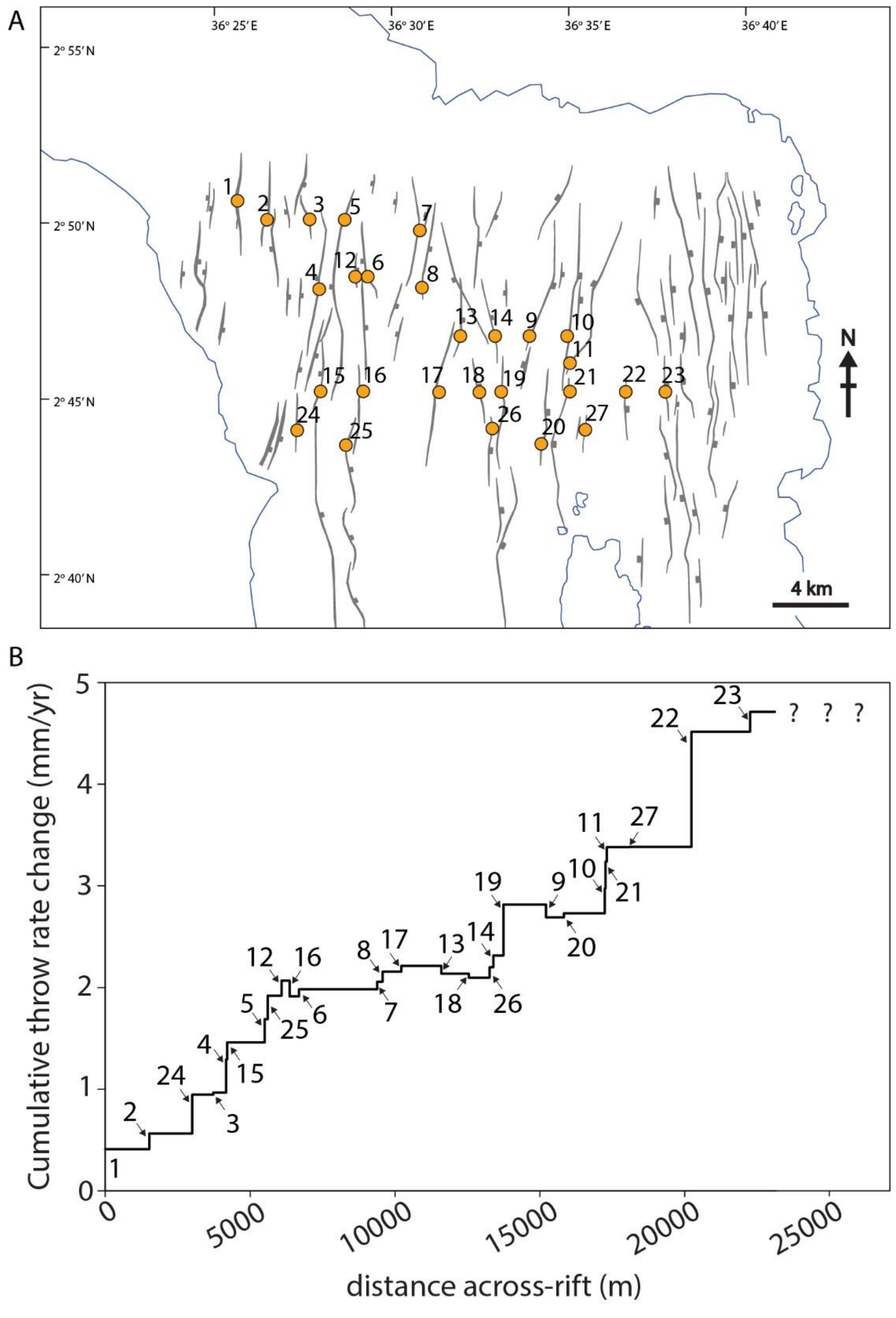

Supplementary Figure S7. A) Position of all 27 faults analysed in this study, with analyses shown in Figures S1-S5. Note that Horizon 2 of Ref[37] cannot be confidently mapped along the eastern edge of the basin, and hence no data were acquired on these faults. B) Across-strike cumulative throw rate from Figure S6 with the position of each fault identified.

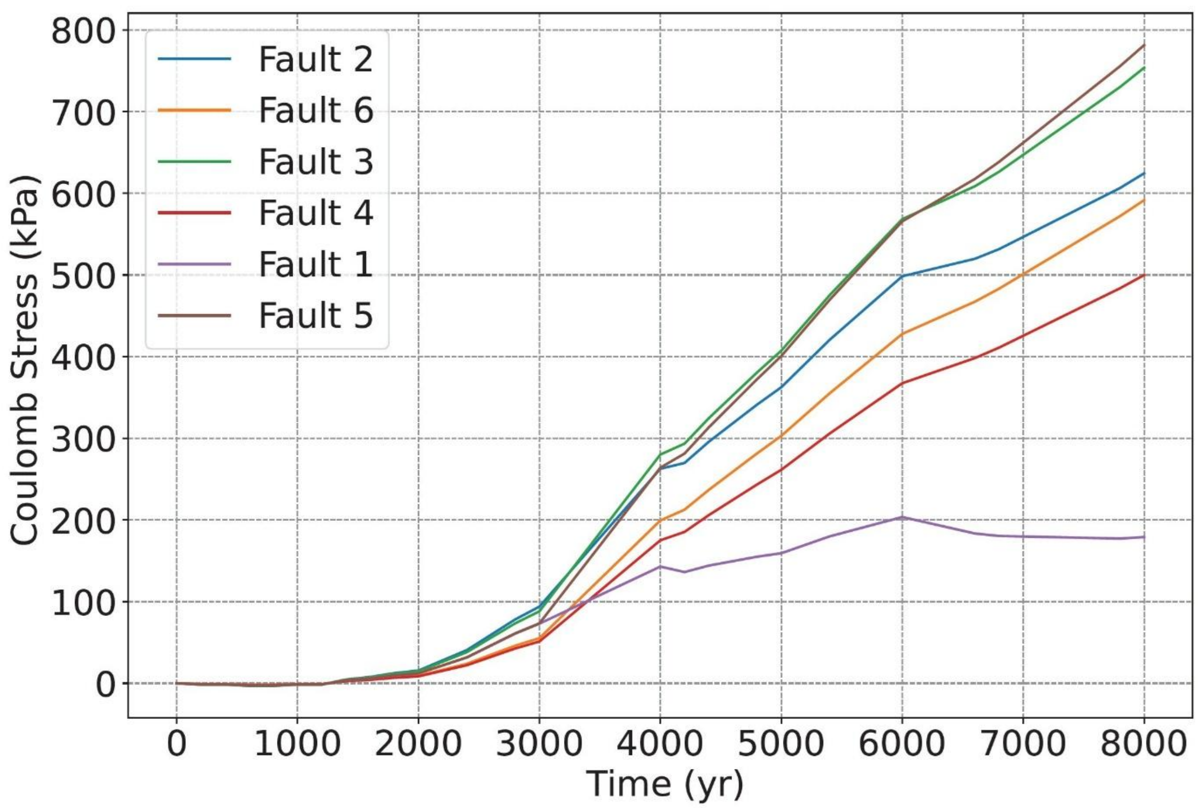

Supplementary Figure S8. Numerical model simulation illustrating the coupled effect of lake unloading and magma inflation on coulomb stress changes for the 6 faults shown in Figure 4.
